# Towards a Deeper Understanding of *Chlamydia trachomatis* Pathogenetic Mechanisms: Editorial to the Special Issue “*Chlamydia trachomatis* Pathogenicity and Disease”

**DOI:** 10.3390/ijms23073943

**Published:** 2022-04-01

**Authors:** Simone Filardo, Marisa Di Pietro, Rosa Sessa

**Affiliations:** Department of Public Health and Infectious Diseases, University of Rome “Sapienza”, P.le Aldo Moro 5, 00185 Rome, Italy; simone.filardo@uniroma1.it (S.F.); marisa.dipietro@uniroma1.it (M.D.P.)

*Chlamydia trachomatis*, an obligate intracellular Gram-negative bacterium, is characterized by a wide range of different serotypes responsible for several local or systemic human diseases, including genital tract manifestations (D–K), trachoma (A–C), and lymphogranuloma venereum (L1–3). Among them, *C. trachomatis* genital infections are the most common sexually transmitted diseases of bacterial origin, with more than 130 million new cases per year worldwide [1]. *C. trachomatis* genital infection is still a relevant public health problem due to the high prevalence of asymptomatic infections, in both women and men (80–90% and 50%, respectively) that, untreated, may lead to chronic complications, such as pelvic inflammatory disease, ectopic pregnancy, as well as reactive arthritis and infertility [2]. The involvement of *C. trachomatis* in these pathologies is related to its ability to infect and reproduce within different cell types beside the epithelial cells of the genital tract, including synovial and testicular cells [3,4,5]. Following chlamydial infection, target tissue activates mechanisms of cell-autonomous immunity, undergoes cellular changes, and produces proinflammatory cytokines, recruiting innate immune cells [6]. As the infection proceeds, antigen-presenting cells, in turn, activate adaptive immunity, leading to the production of anti-Chlamydia antibodies and the migration of Chlamydia-specific CD4 and CD8 T-cells, resulting in an inflammatory environment that frequently clears the infection but also damages the infected tissue [7].

To date, it is of utmost importance to shed light on the pathogenetic mechanisms underlying host–Chlamydia interaction and influencing the clinical outcomes of chlamydial-mediated genital diseases. Over the course of the last decades, as for example, the injection of chlamydial virulence factors in host cells via the type-3 secretion system, the escape from the endocytic pathway via chlamydial Incs proteins, have been described as mechanisms responsible for chlamydial adhesion, invasion and intracellular survival [8]. In recent years, the importance of the multi-faceted interaction between the host and the resident microflora of the female genital tract has also emerged, as a first line of defense against *C. trachomatis* infection [2]. Indeed, several studies have characterized the cervico-vaginal microbiota via metagenomic approaches and advanced statistical algorithms, evidencing networks of specific bacterial species as potential biomarkers of chlamydial genital infection [9]. Furthermore, the information hidden in the 16s rDNA sequencing data have allowed researchers to describe distinct microbial community states of the cervico-vaginal microbiota associated with the risk of acquiring a *C. trachomatis* genital infection. Peculiar cervico-vaginal microbial signatures were also described in *C. trachomatis*-positive pregnant women, or in women after *C. trachomatis* treatment [10,11].

Despite all of this important evidence, the wealth of information from sequencing data is still largely underexploited. It has recently been hypothesized that it is possible to predict metabolic profiles based solely on the sequencing data from a microbial community, favored also by the large amount of metabolomic data deposited in public databases [12]. In this regard, several in silico approaches have been proposed, such as, for example, PICRUSt, the compound prediction of MIMOSA, MelonnPan and others, that can, indeed, provide valuable information on the metabolic potential of a given microbial community and identify the microbial taxa most likely responsible for the synthesis and/or consumption of key metabolites [12]. An example of their application came from Raimondi et al. (2021), where the researchers observed, in the cervico-vaginal environment of *C. trachomatis*-positive patients, a higher involvement of the biosynthesis of chorismate, [13], a precursor of indole and, hence, of tryptophan, recognized as a fundamental component for the growth and pathogenesis of *C. trachomatis*.

Consequently, all these advanced bioinformatic approaches might be of great help for a deeper understanding of the pathogenetic pathways underlying *C. trachomatis* genital infection. Indeed, their application may generate novel, testable hypotheses that will bolster and accelerate future mechanistic studies, as well as the discovery of novel potential targets for drug development. Many critical questions still remain unanswered, such as the double-edged interaction between *C. trachomatis* and the resident microbiota, as well as the nature of the host susceptibility or resistance to this pathogen and the development of innate or adaptive immune responses. Therefore, this Special Issue will serve as a collection of the most up-to-date progress in the field.
